# Severe Rhabdomyolysis With Minimal Muscular Symptoms After Long-Term Rosuvastatin Use: A Case Report and Brief Literature Review

**DOI:** 10.7759/cureus.117283

**Published:** 2026-10-01

**Authors:** Alireza Izadian Bidgoli, Alberto Gomez Usatorres, Hellen Ailette Gomez Usatorres, Alberto Gomez Veliz, Tariq Qayum

**Affiliations:** 1 Internal Medicine, Jackson Memorial Hospital, Miami, USA; 2 Surgery, University of Vermont Larner College of Medicine, Burlington, USA; 3 Surgery, Jackson Memorial Hospital, Miami, USA

**Keywords:** chronic kidney disease, creatine kinase, rhabdomyolysis, rosuvastatin, statin-associated myopathy

## Abstract

Severe rhabdomyolysis may present with limited muscular symptoms despite substantial biochemical injury. We describe a case of suspected statin-associated rhabdomyolysis during prolonged rosuvastatin exposure. A 65-year-old man with advanced chronic kidney disease, poorly controlled type 2 diabetes mellitus, peripheral arterial disease requiring bilateral below-knee amputations, and previous rhabdomyolysis presented with generalized weakness, abdominal discomfort, and left flank pain. He had taken rosuvastatin for more than 10 years. Admission investigations demonstrated creatine kinase of 23,107 U/L, creatinine of 7.80 mg/dL, markedly elevated aminotransferases, and brown urine. Creatine kinase subsequently peaked at 38,747 U/L despite limited muscular complaints. Progressive azotemia and encephalopathy prompted hemodialysis. Rosuvastatin was discontinued, and supportive treatment was provided without immunosuppression. The reported myositis panel was negative, while magnetic resonance imaging (MRI) demonstrated mild, nonspecific muscle edema. By discharge, weakness and encephalopathy had resolved, strength was preserved on focused examination, and creatine kinase had decreased to 1,003 U/L. The patient was advised to avoid all statins. Outpatient hemodialysis, rheumatology follow-up, and serial creatine kinase measurements were arranged, with anti-3-hydroxy-3-methylglutaryl-coenzyme A reductase (anti-HMGCR) antibody testing and muscle biopsy planned if creatine kinase remained persistently elevated after discharge. This case highlights the discrepancy between biochemical severity and muscular symptoms during long-standing statin exposure. Improvement after statin withdrawal and supportive care supported a suspected statin-associated process but did not establish causality or exclude an autoimmune mechanism. Continued clinical and biochemical follow-up was essential to document recovery and determine the need for further investigation.

## Introduction

Rhabdomyolysis results from skeletal muscle breakdown and the release of intracellular constituents into the circulation, with consequences ranging from isolated biochemical abnormalities to acute kidney injury and life-threatening electrolyte disturbances [1]. Its clinical spectrum includes presentations with few muscular complaints, making symptom-based recognition potentially unreliable [1].

Statins are an established cause of muscle injury, encompassing self-limited myalgias, rhabdomyolysis, and the distinct entity of immune-mediated necrotizing myopathy [2]. Evaluation therefore requires the consideration of medication exposure, competing causes of muscle injury, and individual susceptibility rather than attribution based solely on statin use [3]. Differentiating toxic muscle injury from autoimmune disease is particularly consequential because statin-associated immune-mediated necrotizing myopathy involves antibodies targeting 3-hydroxy-3-methylglutaryl-coenzyme A reductase (HMGCR) and generally requires immunosuppressive treatment [2]. Integrating the clinical course with appropriate diagnostic testing is therefore essential when evaluating suspected statin-associated myopathy [3].

The present case illustrates a marked discrepancy between biochemical muscle injury and limited persistent muscular symptoms in a patient with advanced chronic kidney disease and long-standing rosuvastatin exposure. We report this case to highlight the diagnostic importance of recognizing severe rhabdomyolysis despite minimal muscular symptoms, critically evaluating possible statin involvement after prolonged treatment, and preserving diagnostic uncertainty when the evaluation for an autoimmune mechanism remains incomplete at the time of hospital discharge.

## Case presentation

Clinical presentation

A 65-year-old man residing in an assisted living facility presented with two to three days of generalized weakness, abdominal discomfort, and left flank pain. Hospital admission was designated day 0. His medical history included advanced chronic kidney disease, type 2 diabetes mellitus complicated by neuropathy and retinopathy, hypertension, hyperlipidemia, and peripheral arterial disease requiring bilateral below-knee amputations. He was taking rosuvastatin at presentation and reported more than 10 years of treatment. Previous episodes of rhabdomyolysis were documented, but their timing, precipitating factors, and relationship to statin exposure were not established in the available records. Information regarding a family history of rhabdomyolysis or prior evaluation for hereditary myopathy was also unavailable.

At presentation, he reported poorly controlled blood glucose but denied fever, chills, or rigors. During subsequent rheumatologic assessment, he described mild bilateral upper-extremity weakness at admission that resolved during hospitalization. He denied chronic muscle weakness, myalgias, dysphagia, cough, dyspnea, or new skin lesions. His transient weakness and limited muscular complaints contrasted with the marked creatine kinase elevation identified during the admission.

Initial assessment and physical examination

The patient was initially afebrile and hemodynamically stable, with a temperature of 36.6°C, blood pressure of 119/73 mmHg, heart rate of 85 beats/minute, respiratory rate of 19 breaths/minute, and oxygen saturation of 100%. He was alert and without acute distress. Cardiovascular examination revealed a regular rhythm without murmurs, and respiratory examination described either clear or bilaterally diminished breath sounds without increased work of breathing. His abdomen was soft, nondistended, and nontender. Bilateral below-knee amputations were noted, without documented peripheral edema or rash.

Early examinations documented preserved active range of motion and no back tenderness. On the later focused rheumatologic examination, strength was graded 5/5 in both upper extremities and with bilateral hip flexion, although the left hip flexors were described as slightly weaker than the right. There was no active synovitis or cutaneous eruption. This examination occurred after the patient’s initial weakness had improved.

Laboratory and diagnostic testing

Admission investigations demonstrated a creatine kinase concentration of 23,107 U/L, creatinine of 7.80 mg/dL, and blood urea nitrogen of 102-103 mg/dL. The admission note cited a previous creatinine concentration of 4.1 mg/dL. Serum glucose was 318-321 mg/dL, and glycated hemoglobin was 10.4%. Venous blood gas analysis showed a pH of 7.25 and a bicarbonate concentration of 19 mmol/L. The serum anion gap was 17, and beta-hydroxybutyrate was mildly elevated at 0.39 mmol/L. Although the treating team documented mild diabetic ketoacidosis, urine ketones were negative, and the recorded ketonemia was modest.

Initial aminotransferase concentrations were markedly elevated, with aspartate aminotransferase of 656 U/L and alanine aminotransferase of 648 U/L. Alkaline phosphatase was 232 U/L, total bilirubin was 0.8 mg/dL, and hemoglobin was 9.2 g/dL. Urinalysis showed brown, turbid urine with positive blood, proteinuria, glucosuria, and marked bacteriuria; microscopy recorded four erythrocytes and 83 leukocytes. Urine culture subsequently grew resistant *Klebsiella*, characterized in later clinical notes as an extended-spectrum beta-lactamase-producing organism.

Creatine kinase initially decreased to 20,224 U/L before reaching its highest documented concentration of 38,747 U/L on day 3. Subsequent values fluctuated before declining substantially. Aldolase was reported as elevated at 152, although the measurement unit was unavailable in the reviewed records. Autoimmune testing demonstrated an antinuclear antibody titer of 1:80, with negative results for the tested extractable nuclear antigen antibodies and anti-double-stranded DNA. The myositis panel was reported as negative. Anti-HMGCR antibody testing and muscle biopsy were planned if creatine kinase remained persistently elevated after discharge despite statin discontinuation. Admission values, observed inpatient ranges, and discharge laboratory findings are summarized in Table 1.

**Table 1:** Laboratory findings at admission, during hospitalization, and at discharge Day 0 denotes hospital admission, and day 11 denotes discharge. Hospitalization ranges represent the lowest and highest documented values available for review, including admission and discharge measurements. Where multiple admission values were reported, their range is shown. Inpatient and discharge renal indices include measurements obtained after hemodialysis was initiated on day 4 and should be interpreted in that context. The glucose reference interval applies to fasting measurements; fasting status at admission was not established. U/L, units per liter; mg/dL, milligrams per deciliter; g/dL, grams per deciliter; mmol/L, millimoles per liter; µL, microliter; mm/hour, millimeters per hour

Laboratory parameter	Admission (day 0)	Observed range during hospitalization	Discharge (day 11)	Reference range	Unit
Creatine kinase	23,107	1,003-38,747	1,003	55-170	U/L
Creatinine	7.80	2.55-7.80	4.31	0.70-1.30	mg/dL
Blood urea nitrogen	102-103	20-113	27	8-20	mg/dL
Aspartate aminotransferase	656	94-803	94	10-40	U/L
Alanine aminotransferase	648	161-648	161	10-40	U/L
Alkaline phosphatase	232	122-235	122	30-120	U/L
Total bilirubin	0.8	0.6-0.8	0.7	0.3-1.0	mg/dL
Albumin	4.1	2.4-4.1	2.8	3.5-5.5	g/dL
Serum glucose	318-321	82-358	99	70-99 (fasting)	mg/dL
Sodium	134	130-139	132	136-145	mmol/L
Potassium	3.9	2.9-4.0	3.6	3.5-5.0	mmol/L
Chloride	97-98	91-104	93	98-106	mmol/L
Serum total carbon dioxide	19-20	14-35	29	23-30	mmol/L
Anion gap	17	3-20	10	7-13	mmol/L
Total calcium	8.8	7.1-8.8	7.9	8.6-10.2	mg/dL
Phosphorus	9.7	2.9-9.7	5.2	2.5-4.5	mg/dL
Magnesium	2.4	1.6-2.4	1.7	1.6-2.6	mg/dL
White blood cell count	8.7	7.6-15.0	8.1	4.0-11.0	×10³/µL
Hemoglobin	9.2	7.5-9.2	7.7	14-18	g/dL
Erythrocyte sedimentation rate	Not available	50-87	69	0-15	mm/hour

Abdominal computed tomography demonstrated mild diffuse bladder wall thickening and cholelithiasis without hydronephrosis or obstructive uropathy (Figure 1). Ultrasonography showed no biliary ductal dilation. Testing for acute hepatitis A and B and hepatitis C antibodies was negative. Lower-extremity arterial duplex ultrasonography demonstrated no hemodynamically significant stenosis or occlusion in the evaluated vessels.

**Figure 1:**
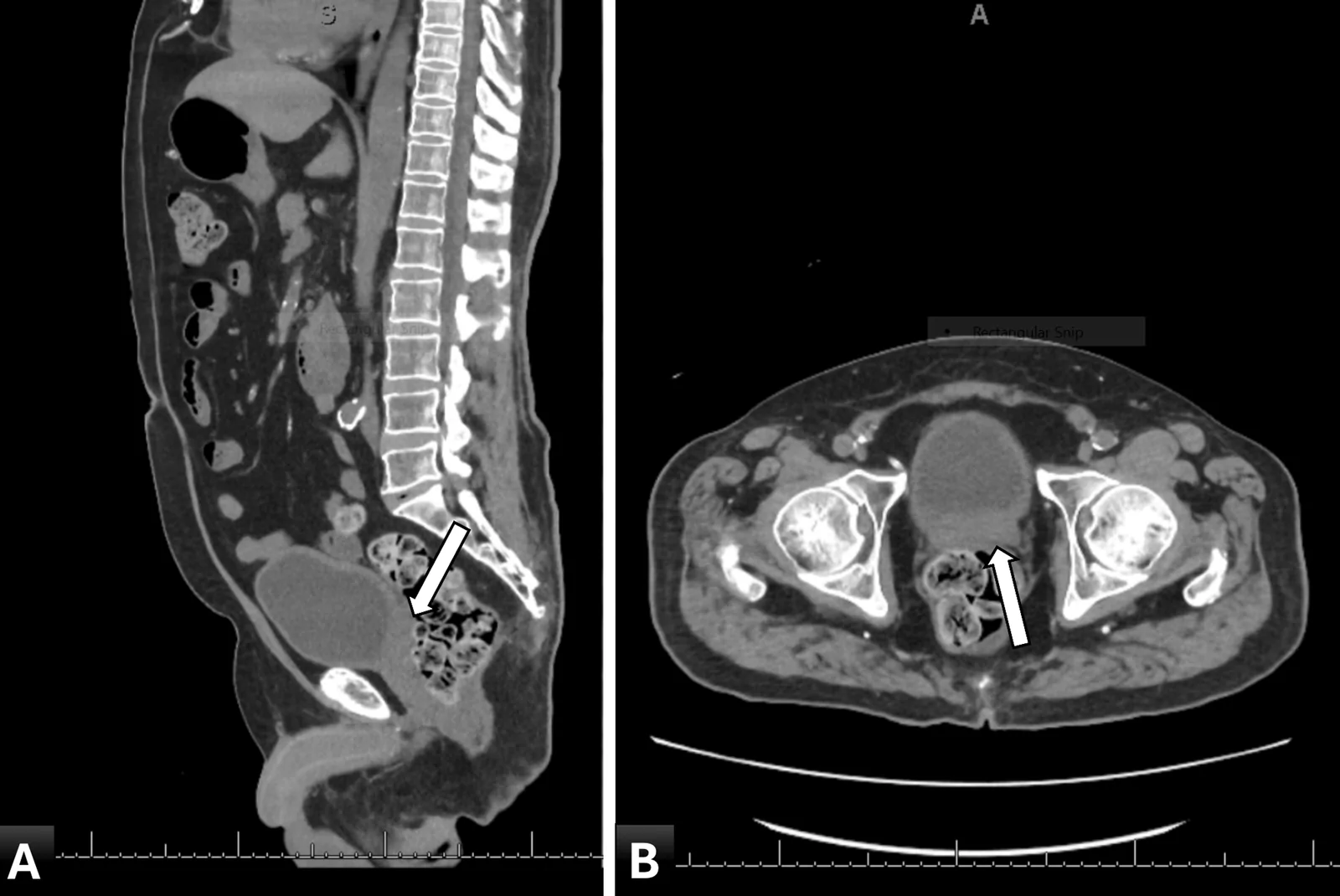
Mild diffuse urinary bladder wall thickening on noncontrast computed tomography Sagittal (A) and axial (B) noncontrast computed tomography images of the abdomen and pelvis demonstrate mild diffuse thickening of the urinary bladder wall (arrows), a nonspecific finding that may be associated with cystitis in the appropriate clinical setting

Noncontrast magnetic resonance imaging (MRI) of the left upper arm, performed near discharge, demonstrated mild homogeneous edema involving the anterior and posterior deltoid, proximal long head of the triceps, rhomboid, and trapezius muscles. The radiologist characterized these findings as nonspecific, noting that they could occur with inflammatory myositis and recommending tissue correlation for definitive diagnosis (Figure 2).

**Figure 2:**
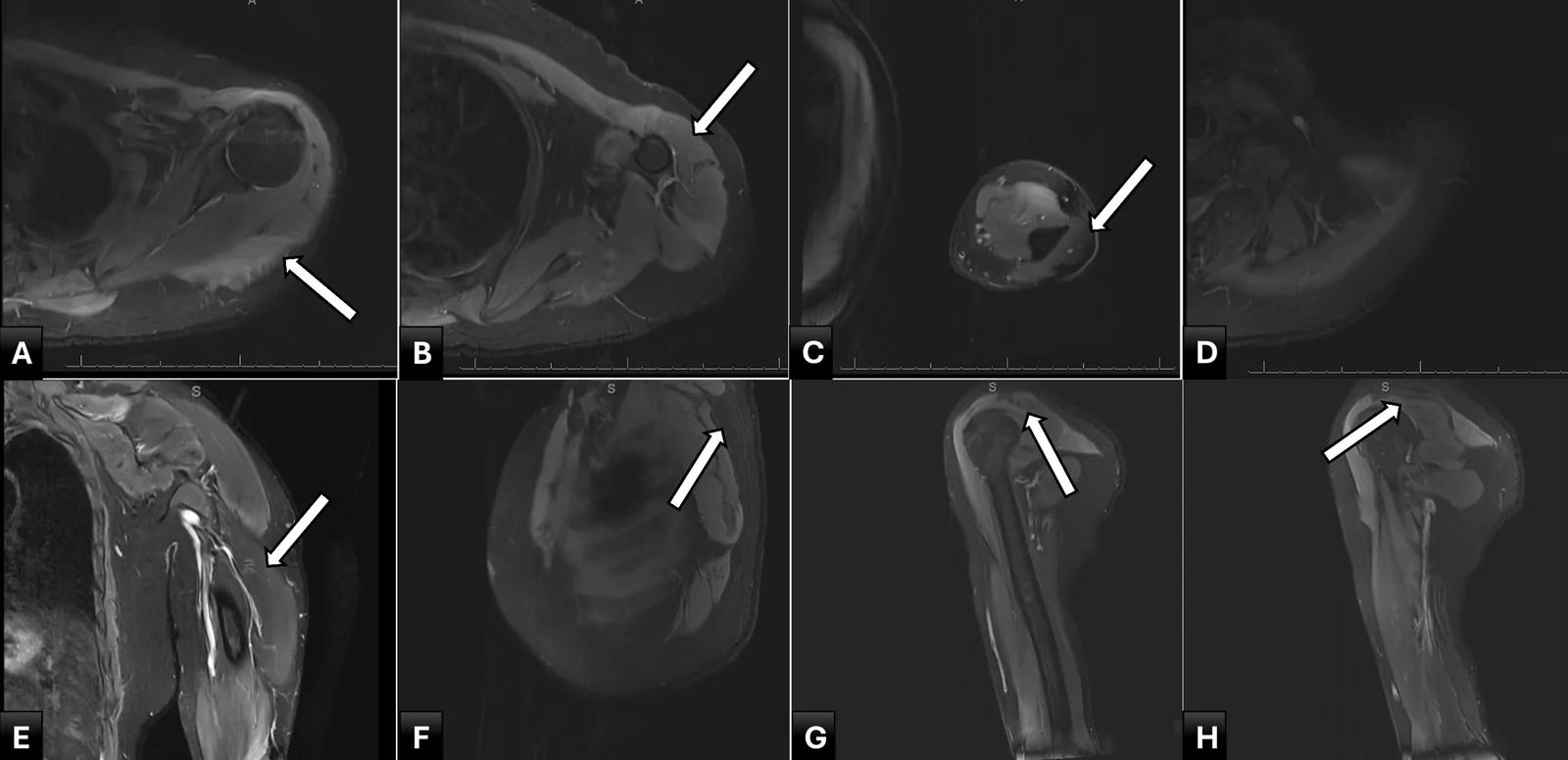
Noncontrast MRI demonstrating mild muscle edema in the left shoulder and upper arm Representative axial (A-D) and longitudinal (E-H) noncontrast magnetic resonance images of the left shoulder and upper arm obtained near discharge. Arrows highlight selected areas of increased signal corresponding to muscle edema described in the radiology report. These findings are nonspecific and do not independently establish inflammatory myositis MRI: magnetic resonance imaging

Multidisciplinary assessment

Multidisciplinary assessment occurred through consultations among the hospital medicine, nephrology, rheumatology, and interventional radiology teams; the available records do not document a formal multidisciplinary board meeting. Hepatology consultation was also requested for the aminotransferase abnormalities.

Nephrology assessed the renal deterioration in the context of advanced chronic kidney disease attributed principally to diabetic nephropathy, with a possible hypertensive contribution and concurrent rhabdomyolysis. Rheumatology evaluated the marked muscle enzyme elevation for an inflammatory or immune-mediated myopathy. Long-standing rosuvastatin exposure, improvement following its withdrawal, and the substantial decline in creatine kinase without immunosuppressive treatment favored a statin-associated process. Nevertheless, the nonspecific MRI abnormalities did not establish the underlying mechanism of muscle injury. Rheumatology considered immune-mediated necrotizing myopathy less likely based on the observed clinical course and recommended outpatient clinical assessment and serial creatine kinase measurements, with anti-HMGCR antibody testing and muscle biopsy to be pursued if enzyme elevation persisted despite statin discontinuation.

Diagnosis and management

The working diagnosis was recurrent rhabdomyolysis, supported by marked creatine kinase elevation from 23,107 U/L at admission to a peak of 38,747 U/L on day 3, together with transient weakness, elevated aldolase, and a documented history of previous episodes. The current episode occurred in the setting of the acute deterioration of advanced chronic kidney disease. Rosuvastatin was considered a potential contributor given ongoing exposure and subsequent biochemical improvement following its withdrawal and supportive care, although this temporal association did not establish causality. Rosuvastatin was discontinued, and the patient was advised to avoid further statin treatment.

Initial treatment included intravenous normal saline at 100 mL/hour, the serial assessment of renal function and muscle enzymes, and insulin for hyperglycemia. Sodium bicarbonate and oral citrate were subsequently administered under nephrology guidance. Progressive azotemia and lethargy prompted transfer to an intermediate-care setting for urgent dialysis access. Hemodialysis was initiated on day 4 through a temporary right internal jugular catheter; a tunneled catheter was placed on day 9.

Empiric ceftriaxone was administered for a suspected urinary tract infection. Following the development of fever and leukocytosis and the identification of resistant *Klebsiella* in the urine culture, treatment was escalated to meropenem. Subsequent clinical assessments favored chronic urinary colonization, and antibiotics were discontinued with continued observation. No immunosuppressive therapy was administered. Rheumatology follow-up and serial outpatient creatine kinase measurements were planned, with anti-HMGCR antibody testing and muscle biopsy to be pursued if enzyme elevation persisted after discharge despite statin discontinuation.

Clinical outcome

The patient’s weakness and encephalopathy resolved, and he reported feeling well by the end of hospitalization. Creatine kinase declined to 11,666 U/L on day 9 and 1,003 U/L on day 11. Aminotransferases also improved, with aspartate aminotransferase and alanine aminotransferase decreasing to 94 U/L and 161 U/L, respectively, on day 11. He denied ongoing myalgias or weakness and demonstrated preserved strength on focused rheumatologic examination. Clinical and biochemical improvement occurred following statin withdrawal and supportive treatment without immunosuppression.

He was discharged in stable condition to his assisted living facility with outpatient hemodialysis and rheumatology follow-up arranged. Discharge recommendations included the continued avoidance of all statins and serial creatine kinase measurements. Persistent enzyme elevation despite statin discontinuation would prompt further evaluation with anti-HMGCR antibody testing and muscle biopsy. Postdischarge laboratory results and clinical outcomes were unavailable; therefore, subsequent creatine kinase normalization, renal recovery, and the need for additional diagnostic testing could not be established.

## Discussion

Background

The modern understanding of rhabdomyolysis developed from observations linking extensive muscle injury to renal failure, particularly Bywaters and Beall’s 1941 description of crush injuries sustained during the London Blitz, although earlier accounts existed [4]. The subsequent recognition of medication toxicity, metabolic disorders, infections, and exertional injury established rhabdomyolysis as a syndrome with diverse traumatic and nontraumatic causes [1].

Statin-associated rhabdomyolysis represents the severe end of a heterogeneous spectrum of muscle adverse effects, with incidence estimates ranging from approximately 0.1 to 8.4 cases per 100,000 patient-years across differing populations and treatment settings [5]. It should be distinguished from nonspecific muscular complaints: a meta-analysis of 19 placebo-controlled trials reported muscle pain or weakness in 27.1% of statin recipients and 26.6% of placebo recipients, indicating that most reported symptoms were not attributable to statins [6].

Susceptibility factors include advanced age, renal or hepatic impairment, hypothyroidism, pre-existing neuromuscular disease, strenuous exercise, and interacting medications [5]. Genetic variation in drug transport may also influence risk, with the association between SLCO1B1 variants and myotoxicity particularly established for simvastatin [5]. Changes in organ function or concurrent medications can modify susceptibility during treatment, making previous tolerance insufficient to exclude subsequent toxicity [7].

The distinction between self-limited toxic injury and immune-mediated necrotizing myopathy depends partly on the clinical and biochemical course after statin withdrawal [2]. Persistent weakness or creatine kinase elevation warrants reassessment for alternative or autoimmune causes, with anti-HMGCR antibody testing and muscle biopsy considered according to the clinical context [2]. This framework supports monitoring recovery after discontinuation while reserving additional investigations for persistent abnormalities.

Pathology and pathophysiology

Rhabdomyolysis results from skeletal myocyte membrane disruption or inadequate energy availability; adenosine triphosphate (ATP) depletion impairs calcium transport, producing intracellular calcium accumulation and the activation of enzymes that damage muscle fibers [1]. Proposed mechanisms of statin toxicity include the disruption of mevalonate-derived products, mitochondrial dysfunction, and oxidative stress, although their relative contributions remain incompletely established [5]. Released myoglobin contributes to renal injury through oxidative tubular damage, vasoconstriction, and cast formation, particularly in hypovolemic and acidic conditions [1].

Anti-HMGCR immune-mediated necrotizing myopathy involves sustained autoimmunity against the statin target, potentially maintained by increased HMGCR expression in regenerating muscle fibers [2]. Histology typically demonstrates necrotic and regenerating fibers with relatively sparse inflammatory infiltrates, although overlapping changes can occur in toxic statin myopathy [2]. MRI-detected edema is similarly nonspecific and cannot independently distinguish these mechanisms [8].

In this patient, improvement following statin withdrawal without immunosuppression supported the working diagnosis of a reversible statin-associated process but did not establish its mechanism. Serial creatine kinase monitoring was therefore planned, with anti-HMGCR antibody testing and muscle biopsy to be pursued if enzyme elevation persisted after discharge.

Comparative analysis with the literature

Published reports demonstrate that rosuvastatin-associated muscle injury can emerge after prolonged exposure, although its presentation varies considerably [7,9]. Hassan et al. described a 77-year-old woman who developed progressive bilateral limb weakness after two years of rosuvastatin treatment and improved following withdrawal [9]. In comparison, our patient’s weakness was transient, and subsequent examination demonstrated preserved strength despite substantial biochemical injury. His presentation therefore illustrates limited persistent muscular manifestations rather than entirely asymptomatic disease. The principal distinction is the discrepancy between the magnitude of muscle enzyme elevation and the clinical findings.

Prolonged treatment also complicates the assessment of medication causality. Niedrig et al. reported severe rosuvastatin-associated rhabdomyolysis during acute hepatic and renal dysfunction, emphasizing how changing organ function can increase susceptibility to toxicity [7]. Advanced chronic kidney disease provided a comparable susceptibility context in our patient. However, the available records did not establish his rosuvastatin dose, a recent dose adjustment, or a specific interaction. Previous episodes of rhabdomyolysis were also insufficiently characterized to determine their relationship to statin exposure. These uncertainties support a diagnosis of suspected rosuvastatin-associated rhabdomyolysis while leaving other contributors unresolved. Previous tolerance should prompt the reassessment of evolving risk factors rather than the exclusion of medication-related injury [5,7].

Diagnostic interpretation requires distinguishing the evidence of muscle injury from the evidence of its underlying mechanism. MRI can demonstrate edema but lacks sufficient specificity to establish inflammatory or autoimmune myopathy without supporting clinical and laboratory findings [8]. Accordingly, the mild edema observed in this patient supported muscle involvement but did not resolve the differential diagnosis. Anti-HMGCR immune-mediated necrotizing myopathy typically produces persistent proximal weakness and elevated creatine kinase despite statin discontinuation [2]. The improvement observed without immunosuppression was less typical of that course and supported continued clinical and biochemical observation. The treating team therefore advised the avoidance of all statins and serial outpatient creatine kinase measurements, with anti-HMGCR antibody testing and muscle biopsy planned if enzyme elevation persisted. This approach is consistent with reassessing persistent abnormalities after withdrawal rather than assigning an autoimmune diagnosis from nonspecific imaging findings alone [2,8].

Management recommendations emphasize the removal of contributing exposures and supportive treatment, with fluid administration adapted to the patient’s renal function and volume status [1,5]. The Eastern Association for the Surgery of Trauma guideline conditionally recommends intravenous fluid resuscitation but acknowledges very low-quality evidence and recommends against routine bicarbonate or mannitol for preventing renal complications [10]. Consensus guidance additionally cautions against excessive fluids and reserves renal replacement therapy for conventional renal indications rather than creatine kinase elevation alone [1]. In our patient, cautious hydration and dialysis for worsening azotemia with encephalopathy reflected the constraints imposed by advanced kidney disease. Because alkali was administered alongside other supportive measures, its independent contribution to recovery could not be determined.

Renal outcomes provide another important comparison. In a French multicenter cohort of 387 intensive care patients with severe rhabdomyolysis, 81.4% developed acute kidney injury, and 26.6% required renal replacement therapy; these estimates reflect a selected critically ill population rather than statin-associated cases specifically [11]. Niedrig et al. also reported continued dialysis despite creatine kinase normalization, illustrating that biochemical muscle recovery and renal recovery may diverge [7]. Our patient similarly experienced substantial muscular improvement while requiring ongoing dialysis, although his advanced baseline kidney disease prevents the attribution of dialysis dependence solely to rhabdomyolysis. Postdischarge assessment was intended to establish whether creatine kinase normalized and whether further etiologic investigation was necessary. Without subsequent results, the duration of biochemical recovery, recurrence risk, renal outcome, and need for additional testing remain undetermined.

What we learned from this case

This case demonstrates that severe biochemical muscle injury can coexist with limited muscular symptoms and preserved strength on subsequent examination. Initial weakness resolved despite substantial creatine kinase elevation, emphasizing the importance of interpreting clinical findings alongside laboratory trends.

Long-standing rosuvastatin exposure remained relevant to the medication assessment. Improvement after withdrawal and supportive care supported suspected statin-associated rhabdomyolysis but did not establish causality. The patient was advised to avoid all statins and undergo outpatient creatine kinase monitoring, with anti-HMGCR antibody testing and muscle biopsy planned if elevation persisted.

Muscular improvement did not establish renal recovery: ongoing dialysis requirements remained difficult to attribute specifically to rhabdomyolysis because of advanced pre-existing kidney disease. Follow-up was therefore essential to document biochemical resolution, reassess renal function, and determine whether further investigation was warranted.

Limitations of the study

This single-patient report cannot establish a causal relationship between rosuvastatin exposure and rhabdomyolysis. Advanced chronic kidney disease complicates attribution because substantial renal dysfunction coexisted with the acute muscle injury, making it difficult to separate a medication-related process from the effects of concurrent illness. The available records did not establish the rosuvastatin dose, its appropriateness for the patient’s renal function, recent changes in exposure, or a specific drug interaction. Accordingly, increased drug exposure could not be demonstrated. Improvement followed both statin withdrawal and supportive treatment, preventing the attribution of recovery to medication discontinuation alone.

The history of previous rhabdomyolysis introduces additional uncertainty. The patient’s age at those episodes, precipitating factors, and concurrent statin use were not documented. It therefore remains unclear whether the current episode represented recurrent statin-associated injury or an underlying susceptibility with another cause. Information regarding familial disease or previous evaluation for hereditary myopathy was unavailable. These gaps limit causal inference and support describing rosuvastatin as a suspected contributor rather than an established cause.

The underlying mechanism also remained unconfirmed. Clinical and biochemical improvement without immunosuppression informed the plan for continued observation, with anti-HMGCR antibody testing and muscle biopsy to be pursued if creatine kinase remained persistently elevated after discharge. Nonspecific MRI findings could not resolve the distinction between toxic and immune-mediated muscle injury. Furthermore, preserved strength on later examination does not establish the absence of weakness earlier in the course.

Finally, advanced pre-existing kidney disease limits the assessment of the renal injury attributable specifically to rhabdomyolysis. Continued dialysis at discharge does not independently establish irreversible rhabdomyolysis-associated kidney injury. The absence of postdischarge laboratory and clinical follow-up prevented the confirmation of creatine kinase normalization, renal recovery, recurrence, or the need for further etiologic investigation.

## Conclusions

This case highlights severe rhabdomyolysis with limited muscular symptoms during long-standing rosuvastatin therapy. Improvement following statin withdrawal and supportive care supported a suspected statin-associated process, but advanced chronic kidney disease and incompletely characterized previous episodes limited causal attribution. The patient was advised to avoid all statins and undergo serial creatine kinase monitoring, with anti-HMGCR antibody testing and muscle biopsy planned if elevation persisted after discharge. Muscular improvement did not establish renal recovery, and postdischarge outcomes remained unavailable. The principal lesson is to integrate clinical findings with biochemical severity while preserving diagnostic uncertainty and arranging follow-up to guide further investigation.
